# Procalcitonin is expressed in osteoblasts and limits bone resorption through inhibition of macrophage migration during intermittent PTH treatment

**DOI:** 10.1038/s41413-021-00172-y

**Published:** 2022-01-27

**Authors:** Anke Baranowsky, Denise Jahn, Shan Jiang, Timur Yorgan, Peter Ludewig, Jessika Appelt, Kai K. Albrecht, Ellen Otto, Paul Knapstein, Antonia Donat, Jack Winneberger, Lana Rosenthal, Paul Köhli, Cordula Erdmann, Melanie Fuchs, Karl-Heinz Frosch, Serafeim Tsitsilonis, Michael Amling, Thorsten Schinke, Johannes Keller

**Affiliations:** 1 Department of Trauma and Orthopedic Surgery, University Medical Center Hamburg-Eppendorf, Hamburg, 20246 Germany; 2 Department of Osteology and Biomechanics, University Medical Center Hamburg-Eppendorf, Hamburg, 20246 Germany; 3 Center for Musculoskeletal Surgery, Charité-Universitätsmedizin Berlin, Berlin, 13353 Germany; 4 Julius Wolff Institute for Biomechanics and Musculoskeletal Regeneration, Charité-Universitätsmedizin Berlin, Berlin, 13353 Germany; 5 Department of Neurology, University Medical Center Hamburg-Eppendorf, Hamburg, 20251 Germany; 6 Berlin Institute of Health, Berlin, 10178 Germany

**Keywords:** Bone, Diseases

## Abstract

Intermittent injections of parathyroid hormone (iPTH) are applied clinically to stimulate bone formation by osteoblasts, although continuous elevation of parathyroid hormone (PTH) primarily results in increased bone resorption. Here, we identified *Calca*, encoding the sepsis biomarker procalcitonin (ProCT), as a novel target gene of PTH in murine osteoblasts that inhibits osteoclast formation. During iPTH treatment, mice lacking ProCT develop increased bone resorption with excessive osteoclast formation in both the long bones and axial skeleton. Mechanistically, ProCT inhibits the expression of key mediators involved in the recruitment of macrophages, representing osteoclast precursors. Accordingly, ProCT arrests macrophage migration and causes inhibition of early but not late osteoclastogenesis. In conclusion, our results reveal a potential role of osteoblast-derived ProCT in the bone microenvironment that is required to limit bone resorption during iPTH.

## Introduction

Bone is a highly dynamic tissue that is remodeled throughout the lifespan. Bone turnover includes the degradation of old or damaged bone and the formation of new bone, which is carried out by two highly specialized cell types — osteoclasts and osteoblasts, respectively.^1,2^ Osteoblasts differentiate from mesenchymal stem cells, whereas osteoclasts form through the recruitment and fusion of macrophages.^2^ If the balance between bone formation and resorption is disturbed, osteoporosis, which is one of the most prevalent bone diseases in the aged population worldwide, may develop.^3–5^

Apart from a recently approved monoclonal antibody neutralizing the Wnt antagonist sclerostin, the only currently available treatment regimen to stimulate bone formation is intermittent daily injections of parathyroid hormone (iPTH).^6^ This strategy appears contradictory, as endogenous PTH activates bone resorption by osteoclasts and thus functions as an essential endocrine regulator of calcium and phosphate levels.^7^ Chronically elevated PTH levels, as observed in hyperparathyroidism, cause a high bone turnover state in which bone resorption exceeds bone formation.^8^ However, iPTH treatment primarily stimulates bone formation and stimulates bone resorption to only a minor extent, resulting in a net increase in bone mass, improved bone microarchitecture and increased mechanical strength.^9,10^

In skeletal tissue, PTH primarily exerts its biological effects through the parathyroid hormone 1 receptor (Pth1r). Among other receptors, this G protein-coupled receptor is expressed in mesenchymal stem cells, osteoblasts and osteocytes but not in osteoclasts.^11–14^ Although the precise molecular mechanism by which iPTH stimulates bone formation is currently not entirely clear, previous studies have demonstrated that iPTH increases the proliferation and differentiation of osteoblasts and their precursors both in vitro and in vivo.^15,16^ Moreover, iPTH has been shown to inhibit osteoblast apoptosis and to activate bone lining cells.^17,18^ In regard to bone resorption, it is now understood that the catabolic effect of PTH is mediated through several mechanisms. First, PTH increases the expression of the chemotactic factor CC-chemokine ligand 2 (CCL2, also called MCP1) in osteoblasts, which results in the attraction and migration of osteoclast precursors (i.e., monocytes and macrophages) to the bone surface.^19^ Second, PTH stimulates the expression of receptor Activator of NF-κB Ligand (RANKL) and decreases the expression of its decoy receptor osteoprotegerin (OPG) in osteoblasts, leading to enhanced osteoclastogenesis and bone resorption.^20^

The classical counterplayer of PTH is the peptide hormone calcitonin (CT). Countless studies have demonstrated that CT, applied at supraphysiologic doses, results in prompt inhibition of osteoclast function.^21,22^ However, as these studies were primarily performed with salmon CT, which exhibits a potency 50-fold higher than that of mammalian CT, the function of endogenous CT remains incompletely understood.^23,24^ In this regard, we were previously able to reveal the endogenous function of CT and its receptor, the calcitonin receptor (CTR), as a powerful regulator of osteoclast-osteoblast crosstalk, primarily inhibiting bone formation through its action on osteoclasts in a sphingosine 1-phosphate (S1P)-dependent manner.^25^

CT is encoded by *Calca*, a gene also coding for its prohormone procalcitonin (ProCT) — a widely used sepsis biomarker — along with the neuropeptide calcitonin gene-related peptide alpha (αCGRP).^26–29^ Through tissue-specific alternative splicing, *Calca* gene transcription results in the synthesis of ProCT in the thyroid gland; ProCT is subsequently cleaved by an endopeptidase to yield mature CT.^30,31^ In contrast to CT, the biological function of ProCT is still unclear, although we previously reported increased bone porosity in aged mice lacking ProCT.^25^ In nervous tissue, alternative splicing of *Calca* results in the synthesis of αCGRP, an essential regulator of bone formation.^26,32,33^ *αCgrp*-deficient mice display osteopenia due to decreased bone formation, whereas bone resorption is not affected.^34,35^ Both ProCT and αCGRP have been shown to signal through the CGRP receptor, which is composed of the calcitonin receptor-like receptor (CLR) in complex with receptor activity modifying protein 1 (RAMP1).^36,37^ Although the functions of CT and αCGRP in bone remodeling are well characterized, the potential role of ProCT in skeletal tissue is unknown. Furthermore, little information regarding the potential interactions of *Calca*-derived peptides with iPTH is currently available.

In our study, we hypothesized that iPTH induces the expression of specific mediators in osteoblasts, which contribute to the limitation of bone resorption during osteoanabolic treatment. In this regard, we identified *Calca* as a novel target gene of iPTH in bone-forming osteoblasts. We show that among the three *Calca*-encoded peptides, iPTH specifically induces the expression of the sepsis biomarker ProCT in osteoblasts, which causes arrest of macrophage migration and osteoclast fusion and thus limits bone resorption during iPTH.

## Results

### PTH induces the expression of ProCT in bone and osteoblasts

**Fig. 1:**
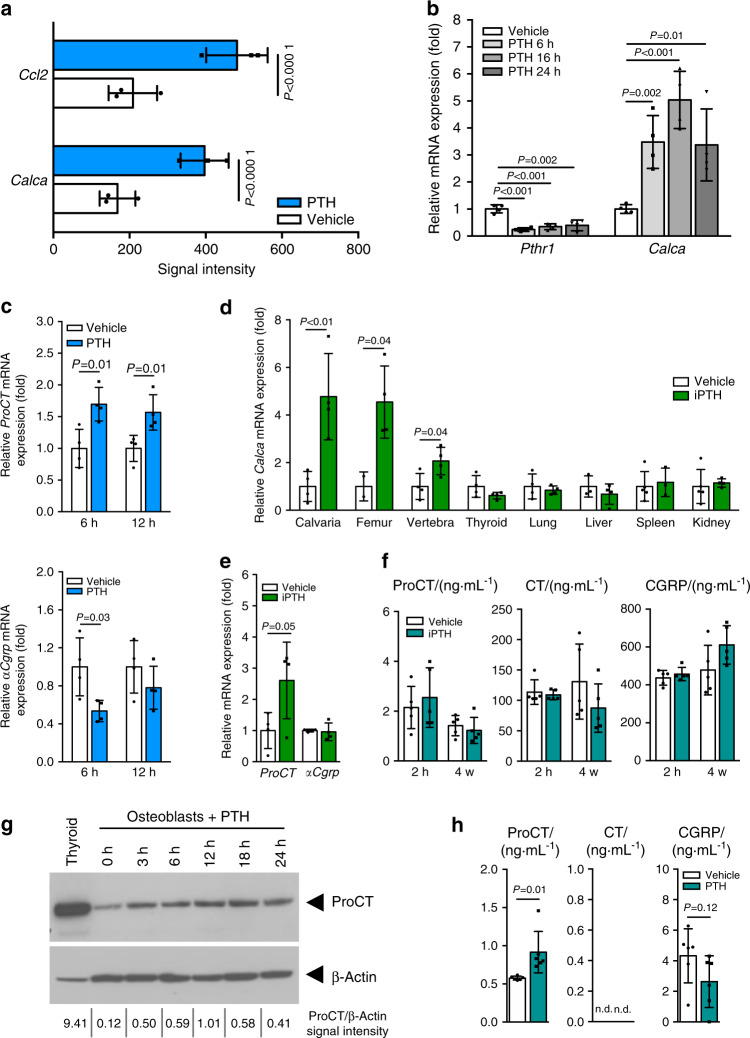
*Calca* is a target gene of PTH in osteoblasts. **a** Affymetrix microarray signal intensities of the indicated genes in calvaria-derived osteoblasts stimulated with PTH (10^−7^ mol·L^−^^1^) for 6 h. *n* = 3 independent experiments per group (Tukey’s biweight mean algorithm). **b** Relative gene expression levels of PTH receptor 1 (*Pthr1*) and *Calca* in calvaria-derived osteoblasts (Day 10 of osteogenic differentiation) following stimulation with PTH. *n* = 4 independent cultures per group (one-way ANOVA followed by Tukey’s post hoc test). **c** Differential expression of *ProCT/CT* and *αCgrp* transcripts (fold) in osteoblasts following 6 and 12 h of stimulation with PTH (10^−7^ mol·L^−1^). *n* = 4 cultures per group (unpaired Student’s *t* test). **d** Relative gene expression levels of *Calca* (fold) in the indicated tissues of mice treated with iPTH (100 μg·kg^−1^) or vehicle for 4 weeks. *n* = 4 mice per group (one-way ANOVA followed by Tukey’s post hoc test). **e** Relative expression levels of the *ProCT/CT* and *αCgrp* transcripts (fold) in femora of the same mice. *n* = 4 mice per group (unpaired Student’s *t* test). **f** Serum concentrations of the indicated proteins in WT mice treated once (serum sampling 2 h after injection) or treated daily for 4 weeks (serum sampling 3–4 h after the last injection) with iPTH or vehicle, as measured by ELISA. *n* = 4-5 mice per group (unpaired Student’s *t* test). **g** Western blot analysis of ProCT expression in calvaria-derived osteoblasts treated with PTH (10^−7^ mol·L^−1^) for the indicated durations. The expected band sizes of ProCT (14 kD; Cloud Clone Corp. antibody) and beta-Actin (45 kD) are indicated. The ProCT/beta-Actin signal intensity ratios are displayed below. **h** Concentrations of the indicated proteins in supernatants of osteoblast cultures stimulated with vehicle or PTH for 6 h. *n* = 6 independent cultures (unpaired Student’s *t* test)

We previously performed genome-wide expression analysis of differentiated osteoblasts in vitro to study the short-term effects of PTH signaling in osteoblasts at the molecular level. We thereby confirmed some of the target genes of PTH established in other studies, including *RANKL* and *CCL2*, but additionally identified yet unknown PTH-induced genes.^38^ Unexpectedly, we also observed that stimulation of osteoblasts with PTH resulted in increased expression of *Calca*, which is usually expressed in nervous and thyroid tissue but not in bone cells (Fig. 1a). We confirmed these findings in independent experiments, where we found that PTH decreased the expression of *Pthr1*, an established target gene of PTH, in osteoblasts and induced *Calca* mRNA expression following 6, 16, or 24 h of stimulation (Fig. 1b). In contrast, *Calca* expression was not significantly induced in a variety of different cells, including D1 mesenchymal stem cells, BV2 microglia, MLO-Y4 osteocyte-like cells, the breast cancer cell line E0771, primary macrophages and HEK cells, indicating that PTH-induced *Calca* induction is specific to osteoblasts (Supplementary Fig. 1a, b). Using primers specific for the *ProCT/CT* and *αCgrp* mRNA transcripts (Supplementary Fig. 2a), we detected increased *ProCT/CT* mRNA levels in osteoblasts stimulated with PTH, but this increase was not observed for the *αCgrp* transcript (Fig. 1c). Analyzing *Calca* expression in wild-type (WT) mice that had been treated with iPTH (daily injection of teriparatide) for 4 weeks, no effects were observed in the thyroid gland, lung, liver, spleen or kidney; however, iPTH treatment led to significantly increased *Calca* expression in skeletal tissues, including the calvaria, femur, and spine (Fig. 1d). Again, we detected increased *ProCT/CT* mRNA levels in the femora of iPTH-treated mice, whereas the *αCgrp* mRNA level was not affected (Fig. 1e). To investigate whether this difference may also affect systemic processes, we measured serum levels of ProCT, CT and CGRP using ELISA; however, we did not observe any alterations in iPTH-treated mice compared to control mice after 4 weeks of daily iPTH (Fig. 1f). Likewise, short-term treatment of mice with iPTH for two hours did not affect the serum levels of *Calca*-encoded peptides. To investigate the effect of increased *Calca* expression at the local level, we performed western blot analysis of osteoblast lysates with two individual antibodies specific for ProCT and included samples obtained at different time points of PTH stimulation and thyroid tissue as positive controls. In addition to the positive signal in thyroid lysates, we observed increased ProCT expression in primary osteoblasts following stimulation with PTH for 3, 6, 12, 18, and 24 h (Fig. 1g) with the first anti-ProCT antibody (Cloud Clone Corp.). With another ProCT-specific antibody (LSBio), we also detected increased ProCT expression in osteoblasts 3, 6, and 14 h following PTH stimulation (Supplementary Fig. 1c). In addition, in supernatants of osteoblast cultures stimulated with PTH for 6 h, we observed increased ProCT levels, as assessed by ELISA (Fig. 1h). Collectively, these results demonstrated that PTH induces the expression of ProCT in osteoblasts.

### Excessive bone resorption in mice lacking ProCT during iPTH

We next aimed to determine the functional relevance of this observation and subjected 12-week-old WT and *Calca*-deficient mice, in which ProCT expression is disabled, to 4 weeks of daily iPTH treatment. As *Calca*-deficient mice also lack CT and αCGRP expression, we additionally tested the potential involvement of these *Calca* splicing products using 2 further mouse lines (Supplementary Fig. 2b). First, we employed our previously described mouse model with global deletion of exons 6 and 7 of CTR (*Calcr*^*−/−*^), in which CT signaling is absent.^25^ Second, we utilized mice carrying a stop codon immediately upstream of the coding region of the *αCgrp* alternative splicing transcript in exon 5 (*αCgrp*^*−/*−^), which disables αCGRP expression but leaves ProCT and CT expression intact.^34^

**Fig. 2:**
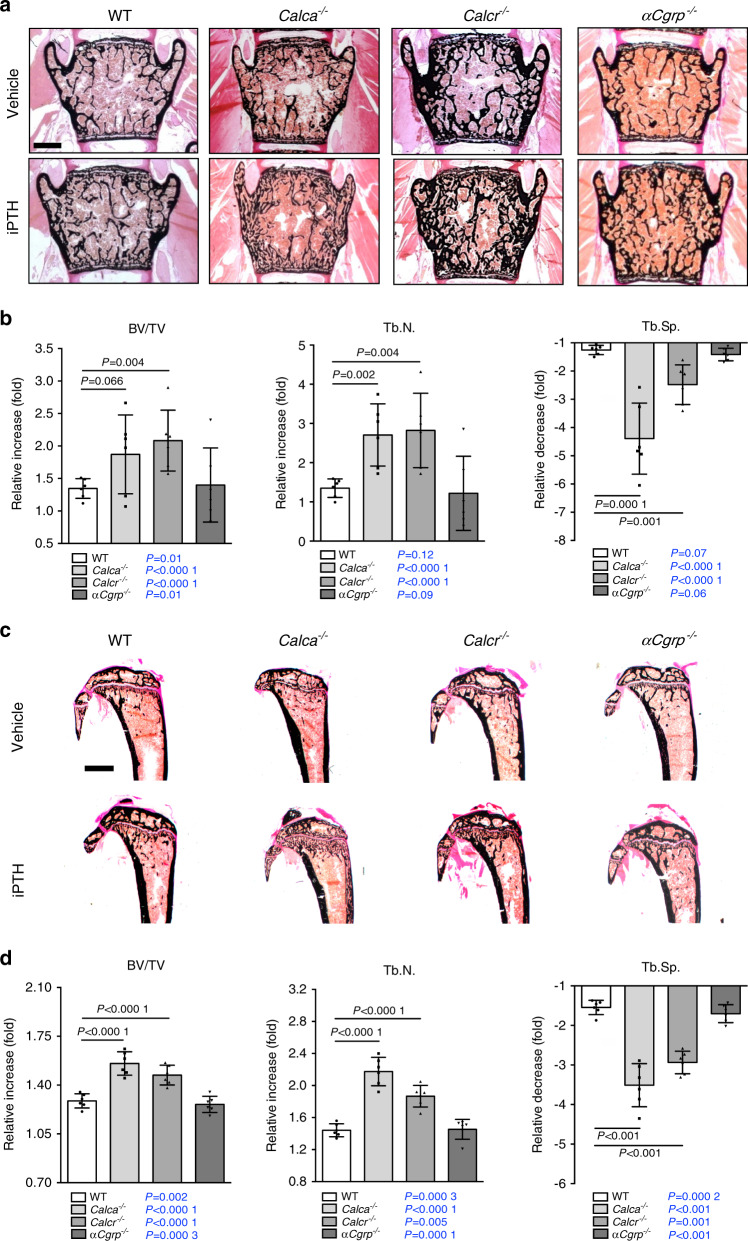
iPTH-induced alterations in bone microarchitecture in *Calca*-deficient mice. **a** Von Kossa/van Gieson staining of undecalcified vertebra sections (L4) from the indicated groups following 4 weeks of treatment with iPTH (100 μg·kg^−1^) or vehicle. Scale bar = 500 μm. **b** Relative alterations (iPTH vs. vehicle) in the bone volume fraction (BV/TV), trabecular number (Tb.N.) and trabecular separation (Tb.Sp.), expressed as fold differences, in the same mice. **c** Von Kossa/van Gieson staining of undecalcified tibia sections from the same mice. Scale bar = 500 μm. **d** Relative alterations (iPTH vs. vehicle) in the bone volume fraction (BV/TV), trabecular number (Tb.N.) and trabecular separation (Tb.Sp.), expressed as fold differences, in the same mice. In (**a**–**d**), *n* = 5-6 mice per group (two-way ANOVA followed by Tukey’s post hoc test). In (**b**, **d**), the *P* values for the statistical comparison between vehicle vs. iPTH treatment in mice of the same genotype are indicated in blue (two-tailed Student’s *t* test)

In WT and *αCgrp*-deficient mice, iPTH treatment resulted in a homogeneous increase in bone mass in the lumbar and thoracic spine, as evidenced by undecalcified histology and μCT scanning (Fig. 2a; Supplementary Fig. 3). In contrast, mice lacking *Calcr* displayed a comparatively greater increase in bone mass, and *Calca*-deficient mice developed a highly irregular bone structure during treatment. As determined by static histomorphometry of spine sections, iPTH caused an increase in trabecular bone volume in all groups (Fig. 2b). In this regard, *Calcr*-deficient mice displayed significantly greater increase than mice in the other groups. Furthermore, iPTH treatment resulted in an increased trabecular number in all treated animals, whereas *Calca-* and *Calcr*-deficient mice showed a significantly more pronounced gain relative to mice in the other groups. Likewise, the relative decrease in trabecular separation compared to that in WT control mice was statistically significant in *Calca*- and *Calcr*-deficient mice, while it was less pronounced in mice lacking αCGRP. In the abovementioned groups, undecalcified tibial sections demonstrated similar morphological changes, including a highly irregular, porous bone structure in iPTH-treated *Calca*-deficient mice (Fig. 2c). Histomorphometric quantification of the proximal tibia showed iPTH-induced changes comparable to those observed in lumbar spine sections, with the exception of the relative increase in BV/TV, which was significantly elevated in both *Calca*- and *Calcr*-deficient mice (Fig. 2d; Supplementary Fig. 4). Of note, the BV/TV in spine and tibia sections was significantly increased by iPTH treatment in mice of all genotypes, as indicated separately (Fig. 2b, d; blue *P* values).

**Fig. 3:**
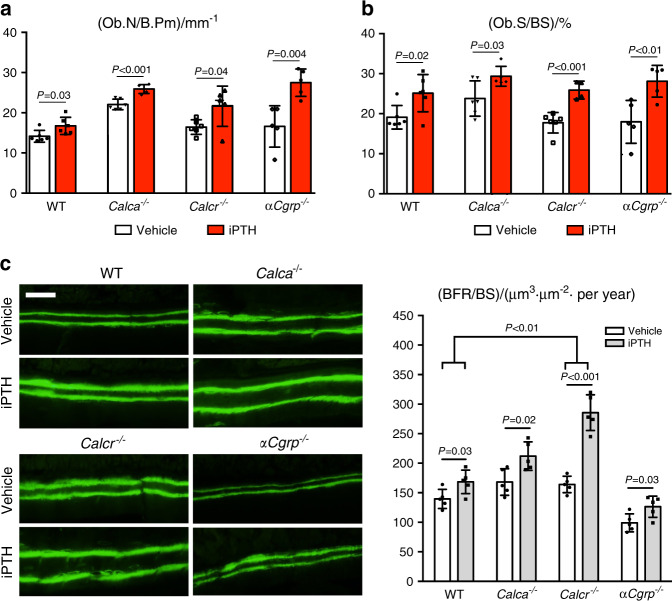
Enhanced anabolic response of osteoblasts to iPTH in mice lacking *Calca* and *Calcr*. **a**, **b** Quantification of the number of osteoblasts per bone perimeter (Ob.N/B.Pm) and osteoblast surface per bone surface (Ob.S/BS) in trabecular bone of spine sections from the indicated groups following 4 weeks of treatment with iPTH (100 μg·kg^−1^) or vehicle. *n* = 5–6 mice per group (one-way ANOVA followed by Tukey’s post hoc test). **c** Representative fluorescence images of double calcein labeling in trabecular bone of spine sections (scale bar = 25 μm) and quantification of the bone formation rate per bone surface (BFR/BS). *n* = 5 mice per group. Comparisons between vehicle- and iPTH-treated mice of each genotype were performed with unpaired Student’s *t* test. Comparisons between the relative alterations in each group were performed with two-way ANOVA followed by Tukey’s post hoc test

To understand these observations at the cellular level, we next analyzed osteoblast parameters and bone formation in lumbar spine sections. Here, we observed that iPTH treatment elevated the osteoblast number to a similar degree in mice of all genotypes (Fig. 3a). Likewise, the osteoblast surface was increased in all experimental groups treated with iPTH compared to the respective untreated control group (Fig. 3b). The bone formation rate, as assessed by double calcein labeling, was also increased in all iPTH-treated groups. However, the treatment-induced relative increase compared to the rate in WT control mice was statistically significant only in *Calcr*-deficient mice (Fig. 3c).

**Fig. 4:**
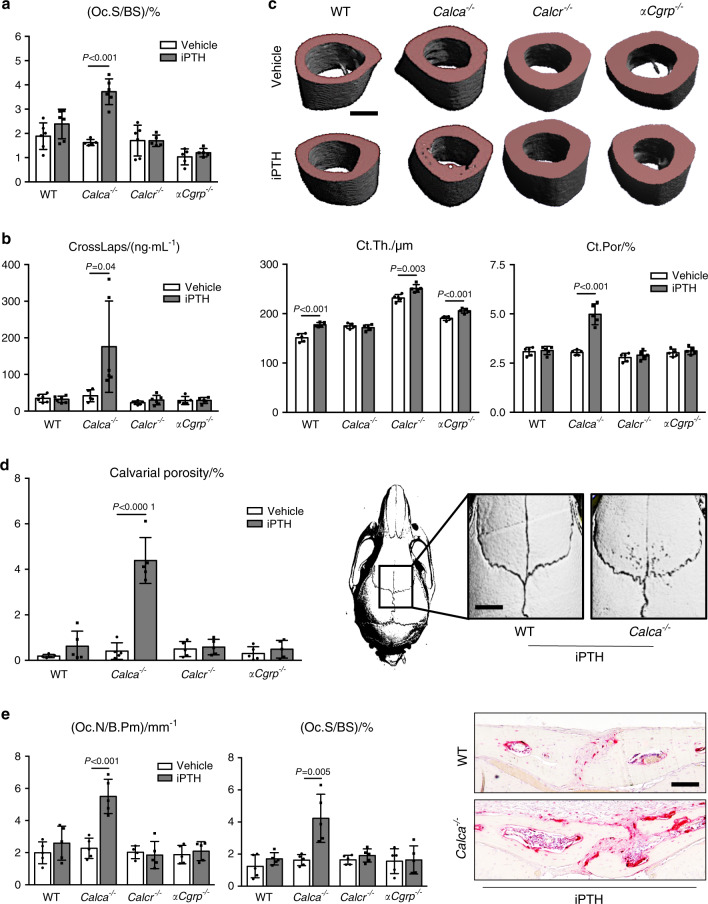
Excessive bone resorption in mice lacking *Calca* in response to iPTH. **a** Quantification of the osteoclast surface per bone surface (Oc.S/BS) in trabecular bone of spine samples derived from the indicated groups following 4 weeks of treatment with iPTH (100 μg·kg^−1^) or vehicle. **b** Serum levels of the bone resorption marker beta-CrossLaps (CrossLaps) in the same mice. **c** Representative μCT images of humeri with quantification of cortical thickness (Ct.Th.) and cortical porosity (Ct.Por) in the same mice four weeks after iPTH treatment (scale bar = 400 μm). **d** Quantification of calvarial porosity in the same mice. Representative μCT images are shown on the right (scale bar = 500 μm). **e** Quantification of the number of osteoclasts per bone perimeter (Oc.N/B.Pm) and osteoclast surface per bone surface (Oc.S/BS) in calvaria of the same mice. Representative TRAP staining images are shown on the right (scale bar = 100 μm). In (**a**–**e**), *n* = 4–6 mice per group as indicated (one-way ANOVA followed by Tukey’s post hoc test)

Assessment of cellular bone resorption parameters showed that iPTH treatment did not affect the osteoclast surface in lumbar spine sections from WT, *Calcr*-, and *αCgrp*-deficient mice (Fig. 4a). In sharp contrast, iPTH treatment was associated with an almost twofold increase in the osteoclast surface in *Calca*-deficient mice. Serum CrossLaps, indicative of osteoclast activity, was significantly elevated during iPTH only in *Calca*-deficient mice and not in any other treatment group (Fig. 4b). μCT scanning of the humerus revealed an increased cortical thickness in all treated groups, with the exception of *Calca*-deficient mice, in which the cortical thickness remained unchanged (Fig. 4c). The opposite pattern was detected for cortical porosity, which was significantly elevated exclusively in *Calca*-deficient mice after iPTH treatment but not in any other group. We next performed μCT scanning of calvarial bones, where we observed that iPTH treatment increased calvarial porosity exclusively in *Calca*-deficient but not in WT, *Calcr*-deficient, or *αCgrp*-deficient animals (Fig. 4d). Likewise, the osteoclast surface in TRAP-stained calvarial sections was significantly increased only in *Calca*-deficient mice and not in any other group (Fig. 4e). To analyze whether the increased bone resorption in *Calca*-deficient mice affects bone quality, we performed biomechanical testing of the femur. In all groups, iPTH treatment resulted in a trend toward increased maximal strength until failure, and this increase was statistically significant in *Calcr*- and *αCgrp*-deficient mice (Supplementary Fig. 5). However, stiffness was reduced exclusively in femora from *Calca*-deficient mice, indicating reduced resistance to mechanical deformation.

### ProCT does not affect osteoblast differentiation or function

**Fig. 5:**
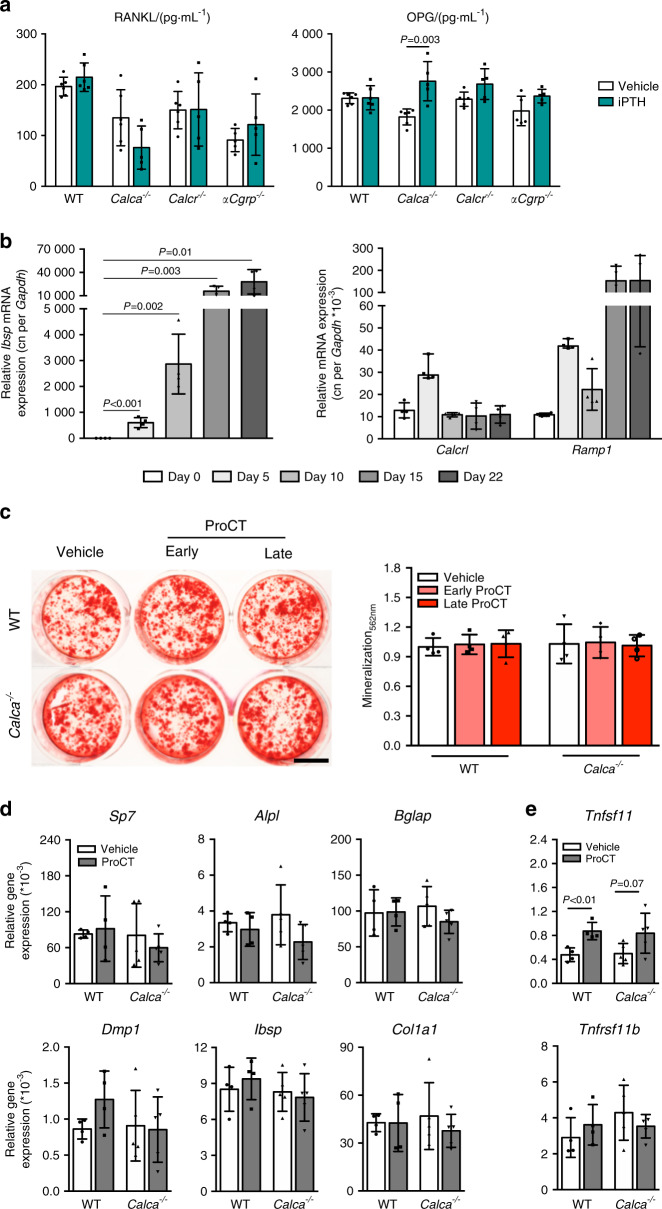
ProCT does not affect osteoblast differentiation or function. **a** Serum levels of proresorptive RANKL and antiresorptive OPG in mice of the indicated genotypes treated with iPTH (100 μg·kg^−1^) or vehicle for 4 weeks. *n* = 5–6 mice per group. **b** Gene expression levels (virtual copy numbers) of the osteoblast marker *Ibsp* as a control (left) and the indicated CGRP receptor components (*Calcrl* and *Ramp1*) in calvaria-derived osteoblasts during the course of differentiation. *n* = 4 independent cultures per group. **c** Alizarin red staining of calvaria-derived osteoblasts (WT and *Calca*^*−/−*^) continuously stimulated with ProCT beginning on Day 0 (ProCT early) or Day 5 (ProCT late) of osteoblast differentiation (scale bar = 4 mm). The quantification of extracellular matrix mineralization is displayed on the right. *n* = 4 independent cultures per group. In (**a**–**c**), one-way ANOVA followed by Tukey’s post hoc test was applied. **d** Relative gene expression levels (fold differences) of the indicated osteoblast markers and (**e**) osteoblast-derived regulators of bone resorption in WT and *Calca*-deficient osteoblasts on Day 10 of differentiation. *Tnfsf11* = RANKL; *Tnfrsf11b* = OPG. *n* = 4-5 independent cultures per group (unpaired Student’s *t* test)

Collectively, these data showed that CTR limits the anabolic response to iPTH, whereas αCGRP is irrelevant for its therapeutic effect. This observation is in line with our previous findings, where we demonstrated that CTR exhibits an S1P-dependent, inhibitory effect on the bone formation rate through its expression in osteoclasts.^25^ In contrast, *Calca*-deficient mice, which lack ProCT, exhibit excessive bone resorption during iPTH treatment. As PTH induces the expression of ProCT primarily in osteoblasts, this observation could potentially be explained by either a modulatory effect of ProCT on osteoblasts that indirectly affects bone resorption or by a direct effect of ProCT on osteoclast formation and function. To test the first possibility, we measured RANKL and OPG levels in serum following iPTH treatment and did not observe significant alterations in any group of mice with the exception of *Calca*-deficient mice, in which increased OPG levels were detected (Fig. 5a). Likewise, in monitoring the effects of short-term iPTH treatment (2 h) on mineral parameters and calciotropic hormones, we observed a tendency toward increased levels of ionized calcium and inorganic phosphate in mice lacking *Calca* and *Calcr* that was not observed in WT or *αCgrp*-deficient mice (Supplementary Fig. 6). Of note, while no alterations in circulating 1,25-dihydroxyvitamin D levels were detected, mice lacking *Calca* and *Calcr* displayed elevated serum concentrations of fibroblast growth factor 23 (FGF23) under baseline conditions, which normalized 2 h after teriparatide injection.

As these observations did not explain the bone resorption phenotype in iPTH-treated mice lacking *Calca*, we next investigated whether ProCT exerts a direct effect on osteoblast differentiation and function in vitro. Although the relevant ProCT receptor, i.e., the CLR–RAMP1 complex, was expressed during osteoblast differentiation (Fig. 5b), ProCT did not affect matrix mineralization when added to calvaria-derived osteoblasts at early (Day 0) and later (Day 5) stages of cell differentiation (Fig. 5c). A comparable lack of impact on osteoblast differentiation was also observed in *Calca*-deficient osteoblasts, which did not exhibit a cell-autonomous defect and showed unchanged extracellular matrix mineralization when treated with ProCT. Moreover, stimulation of WT and *Calca*-deficient osteoblasts with ProCT did not alter the expression of key osteoblast differentiation markers (Fig. 5d) and, unexpectedly, even significantly increased the expression of *Tnfsf11* (encoding the proresorptive protein RANKL) in WT cells (Fig. 5e). Together, these findings ruled out the possibility that ProCT inhibits bone resorption through its action on osteoblasts.

### Osteoblast-derived ProCT inhibits early but not late osteoclastogenesis

**Fig. 6:**
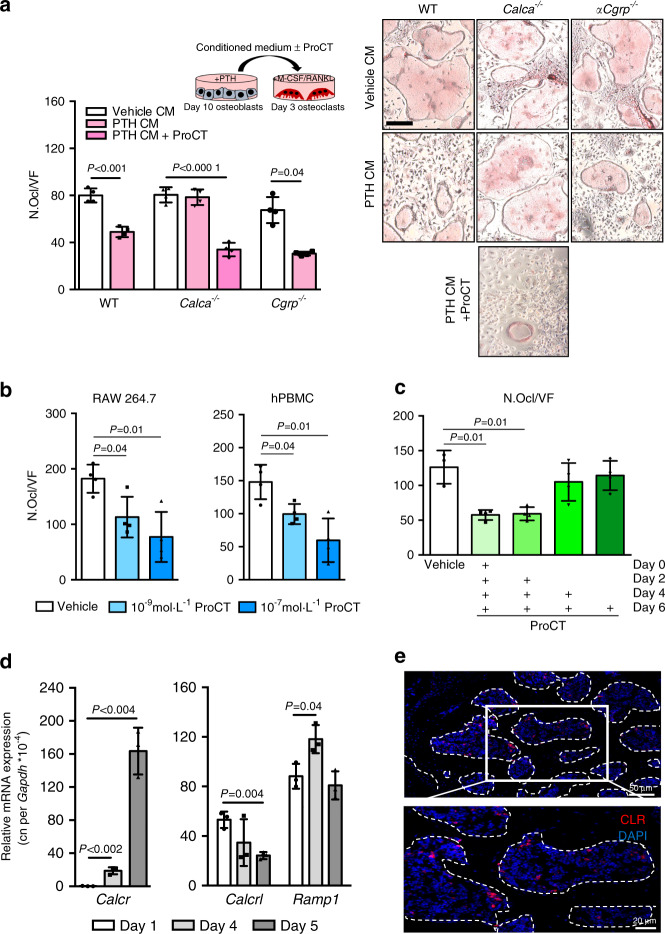
Osteoblast-derived ProCT inhibits early osteoclastogenesis. **a** Representative TRAP staining images of WT osteoclasts on Day 4 of differentiation, after treatment with osteoblast conditioned medium (CM) supplemented with M-CSF, RANKL and/or ProCT (200 ng·mL^−1^) for 24 h (scale bar = 100 μm). CM was collected from calvarial osteoblasts (Day 10 of differentiation) derived from mice of the indicated genotypes and stimulated with iPTH for 24 h. The quantification of osteoclast number per field of view is shown on the left (N.Ocl). *n* = 4 independent cultures per group. **b** Quantification of N.Ocl in the murine monocytic cell line RAW 264.7 or human peripheral blood mononuclear cells (hPBMCs) on Day 5 of osteoclast differentiation. *n* = 4 independent cultures per group. **c** Quantification of the number of TRAP-positive, multinucleated cells (N.Ocl) in murine bone marrow-derived osteoclast cultures continuously stimulated with ProCT (200 ng·mL^−1^) beginning on the indicated days of differentiation. *n* = 3-4 independent cultures per group. **d** Gene expression levels (virtual copy numbers) of the osteoclast marker *Calcr* as a control (left) and the indicated CGRP receptor components (*Calcrl* and *Ramp1*) in murine bone marrow-derived osteoclasts during the course of differentiation. *n* = 3 independent cultures per group. In (**a**–**d**), one-way ANOVA followed by Tukey’s post hoc test was applied. **e** Immunofluorescence staining using a CLR-specific antibody in trabecular bone (spine sections) of untreated WT mice

To test the hypothesis that osteoblast-derived ProCT exerts a direct, inhibitory effect on bone resorption, we stimulated WT, *Calca-*deficient and *αCgrp*-deficient osteoblasts with iPTH and used the conditioned medium from these cells to treat early differentiating WT osteoclasts. Here, conditioned medium from WT and *αCgrp*-deficient osteoblasts resulted in a significant decrease in osteoclast formation, which was not observed for conditioned medium from *Calca*-deficient osteoblasts lacking ProCT (Fig. 6a). In turn, supplementation of conditioned medium from *Calca*-deficient osteoblasts with ProCT resulted in inhibition of osteoclastogenesis. This observation is supported by our previous finding that recombinant ProCT can inhibit osteoclastogenesis in murine bone marrow-derived osteoclasts, independent of CTR.^25^ As this could provide a potential explanation for the increased bone resorption in *Calca*-deficient mice during iPTH treatment, we next confirmed the inhibitory effect on osteoclastogenesis in RAW264.7 cells and human peripheral blood monocytes (hPBMCs), where continuous ProCT treatment beginning with cell differentiation on Day 0 resulted in inhibition of osteoclast formation (Fig. 6b). To further explain these findings, we performed experiments with murine bone marrow cells and found that ProCT inhibited osteoclastogenesis when continuously applied beginning in the early stages (Day 0 and Day 2) of osteoclast differentiation but not when used beginning in the intermediate (Day 4) or late stages (Day 6) (Fig. 6c). This finding was in line with our further observations that the main ProCT receptor component CLR was specifically expressed in osteoclast precursors, i.e., macrophages, with decreasing expression levels during late osteoclastogenesis, whereas RAMP1 was expressed at steady levels (Fig. 6d). Likewise, the CLR protein was detected in mononuclear cells in the bone marrow but not in multinuclear osteoclasts attached to the bone surface (Fig. 6e, Supplementary Fig. 7). Collectively, these results indicated that ProCT acts specifically on macrophages and inhibits the early stages of osteoclastogenesis.

### Inhibition of macrophage migration and osteoclastogenesis through ProCT-dependent transcriptional modulation

**Fig. 7:**
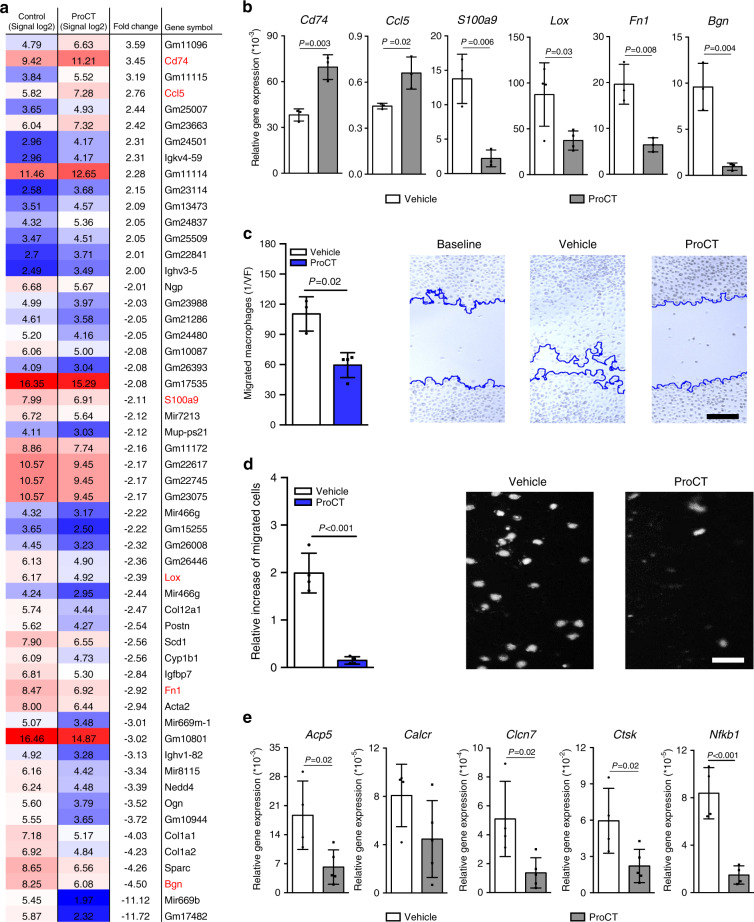
ProCT arrests macrophage migration in vitro. **a** Log signal intensity ratios and relative (fold) expression levels of genes with differential expression in macrophages on Day 7 of differentiation, after treatment with vehicle or ProCT (200 ng·mL^−1^) for 6 h, as determined by genome-wide expression analysis. Pooled mRNA from *n* = 4 independent experiments (Tukey’s biweight average algorithm). The colors in the heatmap indicate absolute signal intensity values (high = red; intermediate = white; moderate = blue). **b** qRT–PCR analysis (virtual copy number per *Gapdh* copy number) of the indicated genes in independent macrophage cultures on Day 7 of differentiation, after stimulation with ProCT (200 ng·mL^−1^) for 6 h. *n* = 3–4 independent experiments. **c** Quantification of migration in primary macrophages treated with vehicle or ProCT for 24 h (scratch assay). Representative images of the cultures are shown on the right, where the blue lines indicate the migration fronts of stimulated cells (scale bar = 100 μm). *n* = 3-4 independent cultures per group. **d** Quantification of macrophage migration across a Transwell membrane toward the chemokine CCL2 during incubation with vehicle or ProCT for 6 h. Representative DAPI staining of the migrated cells is shown on the right (scale bar = 50 μm). *n* = 4 independent experiments per group. **e** qRT–PCR analysis (virtual copy number per *Gapdh* copy number) of the indicated genes in macrophage cultures undergoing osteoclast fusion after stimulation with RANKL (40 ng·mL^−1^) in the presence or absence of ProCT for 6 h (200 ng·mL^−1^). *n* = 4−5 independent cultures per group. For (**b**–**e**), unpaired Student’s *t* test was applied

To understand this effect on a mechanistic level, we next stimulated primary macrophages with ProCT for 6 h and performed genome-wide expression analysis, which allowed us to identify 55 genes with differential expression (Fig. 7a). Among the genes whose expression was increased >2-fold, we identified *Cd74* and Chemokine (C-C motif) ligand 5 (*Ccl5*), both of which have been reported to inhibit osteoclastogenesis in vivo.^39,40^ More interestingly, however, among the genes with a >2-fold decrease in expression, we identified biglycan (*Bgn*), fibronectin (*Fn1*), lysyl oxidase (*Lox*) and S100 calcium-binding protein A9 (*S100a9*), all of which have previously been shown to be of crucial importance in promoting macrophage migration.^41–45^ Therefore, we analyzed gene expression in independent experiments with macrophages treated with ProCT and confirmed the differential expression of these target genes using qRT–PCR (Fig. 7b). To test the hypothesis that the transcriptional regulation induced by ProCT inhibits osteoclast formation through impaired recruitment of precursor cells, we next performed scratch assays to evaluate the migration of bone marrow-derived WT macrophages and observed that ProCT potently inhibited macrophage migration (Fig. 7c). As iPTH treatment was previously shown to attract macrophages and osteoclast precursors to bone remodeling sites by inducing the expression of CCL2 in osteoblasts,^19^ we performed Transwell migration assays and observed that ProCT potently inhibited macrophage migration toward a CCL2 gradient (Fig. 7d). Moreover, macrophages undergoing RANKL-induced cell fusion into osteoclasts showed significantly reduced expression of the key osteoclast markers tartrate resistant acid phosphatase 5 (*Acp5*), *Calcr*, chloride channel 7 (*Clcn7*), cathepsin K (*Ctsk*) and Nuclear Factor Kappa B Subunit 1 (*Nfkb1*) when cotreated with ProCT (Fig. 7e).

### Increased macrophage numbers in the bone marrow of ProCT-deficient mice during iPTH treatment

**Fig. 8:**
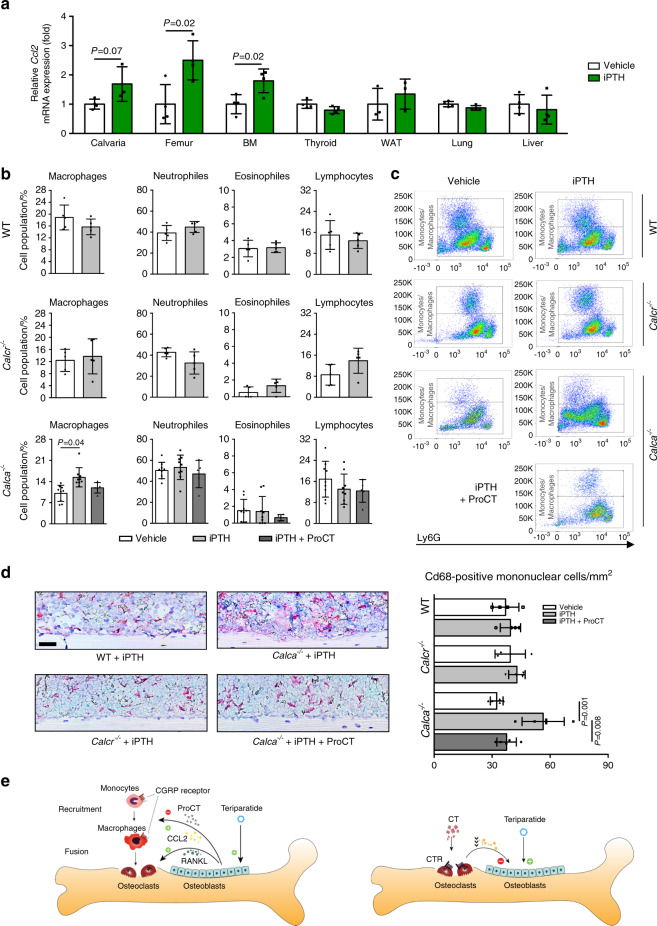
Mice lacking ProCT display increased numbers of macrophages in the bone marrow after iPTH treatment. **a** *Ccl2* expression levels (fold differences) in the indicated tissues following 4 weeks of iPTH treatment (100 μg·kg^−1^). BM = whole bone marrow. *n* = 3-4 mice per group (one-way ANOVA followed by Tukey’s post hoc test). **b** Flow cytometric analyses and (**c**) representative flow cytometry plots of the indicated cell populations in flushed bone marrow from mice of the indicated genotypes following 4 weeks of iPTH (100 μg·kg^−1^) and/or ProCT (10 μg·kg^−1^) treatment. Please also refer to Supplementary Fig. 8 for the gating strategy. *n* = 5–9 mice per group. For *Calca*^*−/−*^ mice, the data were pooled from two independent experiments. **d** Representative images of immunohistochemical staining of tibia sections derived from the same mice using a Cd68-specific antibody. Scale bar = 100 μm. Quantification of intramedullary and endocortical Cd68-positive cells per bone marrow area is displayed on the right. *n* = 4-5 mice per group. In (**b**–**d**), one-way ANOVA followed by Tukey’s post hoc test was applied. **e** Schematic representation of the proposed function of *Calca*-derived peptides in regulating the therapeutic effect of iPTH. Left: iPTH (teriparatide) induces ProCT expression in osteoblasts, which impairs the recruitment of osteoclast precursors (i.e., monocytes and macrophages) to the bone surface and thus inhibits bone resorption. Right: Circulating CT impairs the osteoanabolic effect of iPTH based on tonic inhibition of bone formation, presumably via osteoclasts

We finally confirmed these observations in vivo. In skeletal tissue and in bone marrow of WT mice, iPTH treatment for 4 weeks resulted in the expected increase in the expression of *Ccl2* (Fig. 8a). While no alterations were observed in WT and *Calcr*-deficient mice, iPTH treatment resulted in increased macrophage numbers in the femoral bone marrow of *Calca*-deficient mice compared to vehicle-treated control mice, as assessed by flow cytometry (Fig. 8b, c; for the gating strategy, please see Supplementary Fig. 8). Other cell populations, including those of neutrophils, eosinophils, total lymphocytes, B-cells, T-cells and γδT-cells, were not affected (Supplementary Fig. 9). The increase in macrophage numbers in *Calca*-deficient mice was blunted when ProCT was systemically administered once daily in combination with iPTH. In line with these observations, immunohistochemical staining demonstrated increased numbers of Cd68-positive mononuclear cells in the tibial bone marrow of iPTH-treated *Calca*-deficient mice (Fig. 8d). This increase indicated enhanced macrophage recruitment in *Calca*-deficient mice treated with iPTH, which again was not observed with coadministration of ProCT. Collectively, these results suggest a crucial role of ProCT in the regulation of macrophage recruitment and bone resorption during iPTH treatment (Fig. 8e).

## Discussion

Osteoporosis is the most prevalent bone disease worldwide.^4,5^ Although several agents to inhibit bone resorption are currently available, iPTH is one of the few clinically approved substances for stimulating bone formation.^6^ Application of iPTH is limited to a duration of 2 years, after which the levels of bone resorption markers increase and no net gain in bone mass is achievable.^46–48^ In our current study, we show that *Calca*-encoded peptides are of crucial importance in regulating the therapeutic effect of iPTH. First, mice in which ProCT expression is disabled displayed excessive bone resorption during iPTH treatment. Second, mice with inactivation of the CT/CTR signaling axis due to CT or CTR deficiency exhibited enhancement of the osteoanabolic response to iPTH in regard to bone formation.

Using genome-wide expression analysis, we identified *Calca* as a novel target gene of PTH in osteoblasts. This finding was confirmed in independent experiments at the mRNA and protein levels, which showed that among the three *Calca*-encoded peptides, PTH specifically induces the expression of ProCT. These findings are indeed surprising, as *Calca* expression in healthy organisms is tightly restricted to C cells of the thyroid gland and to neurons of the central and peripheral nervous systems.^28,30,32,49,50^ To understand the full implications of this phenomenon, we studied the impact of iPTH treatment in mice lacking *Calca*. As this genetic model exhibits inactivation of ProCT, CT, and αCGRP signaling and as no mouse model with exclusive deficiency of ProCT currently exists, we evaluated *Calcr*- and *αCgrp*-deficient mice as controls.^25,34,51^ At the age of 3 months, which was employed herein, *Calca*- and *Calcr*-deficient mice are characterized by normal bone resorption and increased bone formation, which is explained by the inhibitory effect of systemic CT on the secretion of osteoanabolic S1P from osteoclasts.^25^ In contrast, *αCgrp*-deficient mice at this age display decreased bone formation with normal bone resorption.^35^ In our study, 4 weeks of iPTH treatment resulted in high bone turnover with elevated bone formation and excessive bone resorption in *Calca*-deficient mice, in contrast to WT mice, which only displayed increased osteoblast parameters. In particular, as we observed normal bone resorption in *Calcr*- and *αCgrp*-deficient animals upon iPTH treatment, these in vivo experiments allowed us to conclude that the excessive bone resorption in *Calca*-deficient mice is indeed caused by the absence of ProCT and not by the lack of CT or αCGRP.

Although several studies have reported potential biological functions of ProCT, a detailed physiological understanding of ProCT is lacking. To date, it has been reported that intracerebroventricular administration of ProCT in rats induces a significant decrease in food intake and weight over a period of >48 h.^52^ Moreover, ProCT has been shown to induce the expression of proinflammatory cytokines in leukocytes.^53^ Our data herein indicate that ProCT exerts a direct inhibitory effect on early osteoclast formation. Of note, due to the lack of a conditional *Calca*-deficiency model, we cannot completely rule out the possibility that other *Calca*-expressing cell types in addition to osteoblasts may also be involved. Therefore, future studies are warranted to test this possibility using models with cell type-specific deficiency. However, as iPTH did not affect the systemic ProCT level, and induction of ProCT expression was exclusively observed in skeletal tissue and osteoblasts, our data suggest that *Calca* expression in osteoblasts is indeed most relevant for the observed effects. Interestingly, we observed increased FGF23 serum levels in mice lacking *Calca* and *Calcr* under standard conditions, and these levels normalized 2 h after teriparatide injection. Although serum concentrations of inorganic phosphate were only slightly affected in *Calca-* and *Calcr*-deficient mice, these findings point toward a yet unknown impact of CT/CTR signaling—but not ProCT signaling—on FGF23 expression, possibly mediated through CTR in osteocytes.^54^ However, although the impacts on FGF23 and phosphate metabolism require further investigation, they do not explain the bone resorption phenotype we observed in *Calca*-deficient mice treated with iPTH. In this regard, on a mechanistic level, we observed that ProCT primarily affected the migratory capacity of macrophages, representing osteoclast precursors. Employing genome-wide expression analysis, we discovered that ProCT induced the expression of established osteoclast inhibitors, including *Cd74* and *Ccl5*. CD74, a type II transmembrane protein that can act as a receptor for macrophage migration inhibitory factor (MIF), was shown to inhibit osteoclast formation in vivo.^39^ Similarly, mice lacking CCL5 have been demonstrated to exhibit increased osteoclastogenesis.^40^ Most importantly, however, ProCT reduced the expression of key mediators of macrophage recruitment, including *Lox*, *Bgn*, *Fn1*, and *S100a9*. LOX has been reported to function as a potent macrophage chemoattractant via activation of the β1 integrin-PYK2 pathway in macrophages.^44^ Biglycan and fibronectin have been demonstrated to regulate macrophage migration and recruitment in different pathophysiologic settings.^41–43^ S100A9 plays an important role in leukocyte migration and is involved in the transepithelial migration of macrophages, thus controlling osteoclastogenesis.^45,55–58^ In particular, release of S100A9 has been suggested to facilitate monocyte and neutrophil transmigration^55,56^ through enhanced expression of the β2 integrin CD11b.^59,60^ In this regard, S100A9 has been shown to induce the differentiation of monocytes toward osteoclasts in vitro, and S100A9-induced osteoclast generation is considered an important reason for bone degradation in infectious osteomyelitis.^57^

On a functional level, the alterations in gene expression translated into a decreased migratory capacity of macrophages, as observed in both the scratch assay and CCL2-directed Transwell migration assay. Of note, as the employed primary macrophage cultures are rather heterogeneous in nature, possible interactions of macrophages with other bone marrow cell types and the potential impact of these interactions on migratory function cannot be excluded in this study. In regard to the immunohistochemical analysis of tibia sections, we observed increased macrophage numbers in the bone marrow of *Calca-*deficient but not *Calcr*-deficient mice during iPTH treatment, highlighting the inhibitory effect of ProCT on macrophage migration also in vivo. It is important to state that osteoclasts also express Cd68. Thus, we excluded Cd68-positive cells from the analysis when they were multinucleated and directly attached to the bone surface. Mechanistically, our findings are supported by research in the sepsis field, where ProCT has been reported to inhibit leukocyte trafficking and recruitment.^61^ While this mechanism may be involved in some of the experimentally reported deleterious effects of ProCT on sepsis progression,^28,62^ it also provides a plausible explanation for the inhibitory effect of ProCT on early osteoclast formation. Thus, although ProCT is primarily known as a sepsis biomarker, our data reveal a novel role in the bone microenvironment that is essential for the therapeutic effect of iPTH.

In terms of bone formation, we observed significantly enhanced osteoblast function in iPTH-treated *Calca*- and *Calcr*-deficient mice compared to iPTH-treated WT control mice. This observation is most likely explained by the absence of CT signaling in both models, as we were previously able to show an inhibitory effect of CT on bone formation.^51^ Thus, it is conceivable that thyroid gland-derived CT produces tonic inhibition of bone formation, which limits the osteoanabolic response to iPTH. In sharp contrast, our data further show that the neuropeptide αCGRP, which exerts an osteoanabolic effect during physiologic bone remodeling, is irrelevant for the therapeutic effect of iPTH in osteoblasts.

This study has several limitations. First, we employed only female mice and are unable to predict whether the same effects will also be observed in male mice. Second, the selected dosage of 100 μg·kg^−1^ per day is in the upper range described in previous studies employing iPTH. Thus, it is possible that the phenotypic manifestations following iPTH treatment may differ in each mouse line at lower concentrations.

Taken together, our study demonstrates that *Calca* signaling controls the therapeutic effect of iPTH. First, we show that the osteoanabolic effect of iPTH is potentiated in the absence of CT and CTR signaling. Second, our data indicate that ProCT is required to limit bone resorption and cortical porosity during iPTH therapy, at least in female mice. We propose that the osteoanabolic effect of iPTH is limited through CT in the systemic circulation, whereas the proresorptive effect of iPTH is controlled by osteoblast-specific expression of ProCT, which impairs the recruitment of osteoclast precursors.

## Methods

### Mice

*Calca*^*−/−*^, *αCgrp*^*−/−*^, and *Calcr*^*−/−*^ mice and WT control mice (all lines on a pure C57BL/6 J background) were genotyped as described.^25,34,51^ For all in vivo experiments, 12-week-old female mice were used. Intraperitoneal iPTH (recombinant human PTH 1–34, Bachem) was administered either as a single administration or daily for 4 weeks, including on weekend days (between 8 and 9 am). Serum was collected 2 h (for the single iPTH treatment) or 3–4 h (for the daily 4-week treatment schedule) after injection. The daily dose of 100 μg·kg^−1^ body weight has been employed in several previous studies, where it was not accompanied by toxic side effects or severe hypercalcemia.^63–65^ In selected cases, recombinant human ProCT (R&D Systems, 10 μg·kg^−1^ body weight) was coadministered daily with iPTH. All in vivo mouse experiments were performed in accordance with the current recommendations of the “Report of the American Veterinary Medicine Association Panel on Euthanasia”. All experimental procedures were performed with approval from the “Behörde für Justiz und Verbraucherschutz-Lebensmittelsicherheit und Veterinärwesen” (G11/024, G09/060, N048/2021) and the “Landesamt für Gesundheit und Soziales, Berlin” (G0285/17).

### Skeletal analysis

Mice were injected twice with calcein (50 mg·kg^−1^ body weight) 9 and 2 days before sacrifice for quantification of the bone formation rate by dynamic histomorphometry. The excised skeletons were fixed overnight with 4% formalin and were then transferred into 80% ethanol. μCT scanning was performed at a voxel resolution of 10 µm using a Scanco μCT 40 (Scanco AG, Switzerland) with settings of 55 kV, 145 μA and a 200 ms integration time. Cortical bone was delineated and evaluated automatically using the image analysis algorithm provided by the manufacturer. The specific region used for analysis of cortical thickness in humeri was a 1-mm thick section of the central shaft distal to the deltoid tuberosity. The trabecular compartment of the 12th thoracic vertebral body was manually segmented, and the trabecular bone microarchitecture was evaluated using the integrated analysis algorithm. For undecalcified histology, bones were dehydrated through ascending concentrations of alcohol before they were embedded in methylmethacrylate. Sections of 4 μm thickness were cut in the sagittal plane using a Microtec rotary microtome. These sections were subjected to von Kossa/van Gieson (for static histomorphometry) and toluidine blue (for cellular histomorphometry) staining procedures. Static, cellular and dynamic histomorphometry at trabecular bone surfaces was carried out according to the guidelines of the American Society for Bone and Mineral Research using an OsteoMeasure system (Osteometrics Inc., USA). TRAP staining was performed on decalcified sections using naphthol AS-MX phosphate (Sigma) and Fast Red Violet LB salt (Sigma) in 40 mmol·L^−1^ acetate buffer (pH 5).

### Cell culture

Primary osteoblasts were isolated by sequential collagenase digestion from calvariae of 5-day-old mice and differentiated for 10 days in the presence of 25 μg·mL^−1^ ascorbic acid and 5 mmol·L^−1^ β-glycerophosphate. To monitor the effects of PTH and ProCT on calvaria-derived osteoblasts, differentiated cells were stimulated with 10^*−*7^ mol·L^−1^ PTH (recombinant human PTH 1–34, Bachem) or 10^*−*7^ mol·L^−1^ ProCT (recombinant human ProCT; R&D Systems) for the indicated durations. Primary osteoclasts were generated as described previously.^25^ In brief, osteoclast precursor cells were isolated from the bone marrow of 12-week-old mice and differentiated for 4 days in α-MEM containing 10 nmol·L^−1^ 1.25(OH)_2_ vitamin D_3_. For the next 6 days, the mice additionally received M-CSF (20 ng·mL^−1^; Peprotech) and RANKL (40 ng·mL^−1^; Peprotech) to allow terminal differentiation. In some cases, the osteoclast medium contained 50% conditioned medium from osteoblasts cultured either in the absence or presence of 10^*−*7^ mol·L^−1^ PTH for 24 h as indicated. Primary macrophages were generated from bone marrow cells cultured with M-CSF (80 ng·mL^−1^; Peprotech) for 7 days. HEK cells, D1 mesenchymal stem cells, BV2 microglia, MLO-Y4 osteocyte-like cells and the breast cancer cell line E0771 were stimulated with PTH for 6 h. RAW 234.7 cells were cultured at a density of 1 × 10^4^ cells per well and were allowed to adhere overnight. The medium was then replaced, and the cells were treated with 5 nmol·L^−1^ (100 ng·mL^−1^) RANKL and/or 200 ng·mL^−1^ ProCT. On Day 5, cultures were stained for TRAP. For generation of osteoclasts from human blood, buffy coat-derived PBMCs were plated in 96-well plates with α-MEM + 10% FCS (1 × 10^6^ cells/well). Two hours later, the plates were rinsed with medium to remove nonadherent cells, and the remaining cells were then cultured for 2 days in α-MEM/FCS supplemented with M-CSF (50 ng·mL^−1^) to generate osteoclast precursors. Osteoclast precursors were further stimulated with M-CSF (50 ng·mL^−1^) and RANKL (50 ng·mL^−1^) in α-MEM/FCS and stained on Day 7 for TRAP. Unless stated otherwise, the term “independent cultures” in the figure legends refers to the number of experiments performed with cells derived from independent mice.

### Cellular assays

TRAP staining was performed as described previously.^25^ In brief, after removal of the medium and two washing steps with phosphate-buffered saline (PBS), cells were fixed with cold methanol for 5 min. After washing and drying, cells were stained with naphthol AS-MX-phosphate (Sigma) and Fast Red (Sigma) for 30 min before the number of TRAP-positive multinuclear cells per well was determined. For alizarin red staining, cells were incubated with 40 mmol·L^−1^ alizarin red staining solution (pH 4.2) for 10 min at room temperature after fixation with 90% ethanol. After additional washing steps with distilled water, cell-bound alizarin red was dissolved in 10% acetic acid. After incubation for 30 min at room temperature, the absorbance was measured at 562 nm.

For the scratch assay, bone marrow-derived macrophages were cultured to 95% confluence. After two hours of stimulation with 10 ng·mL^−1^ LPS for macrophage activation in the presence or absence of 200 ng·mL^−1^ ProCT (R&D Systems), a 600–800 µm scratch was made in the monolayer. Subsequently, the cells were washed, while ProCT remained in the assay media, and migrated macrophages were counted after 24 h. For the Transwell migration assay of bone marrow-derived macrophages, cells were prestimulated for 1 h with 10 ng·mL^−1^ LPS, detached and then seeded in Boyden chambers with a 5 µm pore size in medium containing 200 ng·mL^−1^ ProCT or vehicle. The lower chamber contained the chemoattractant CCL2 (200 ng·mL^−1^; Peprotech), and the migration of cells across the membrane was evaluated after 6 h.

### Expression analysis

RNA was isolated using an RNeasy Mini Kit (Qiagen), and DNase digestion was performed according to the manufacturer’s instructions. The concentration and quality of RNA were measured using a NanoDrop ND-1000 system (NanoDrop Technology). For expression analysis, 1 μg of RNA was reverse transcribed using a Verso cDNA Synthesis Kit (Thermo Fisher Scientific) according to the manufacturer’s instructions. Quantitative real-time PCR (qRT–PCR) was carried out using predesigned TaqMan primers. To differentiate between *ProCT/CT* and *αCgrp* transcripts, qRT–PCR was performed with Power SYBR Green PCR Master Mix (Sigma Aldrich) with the following primer sequences: *ProCT/CT* FOR AGGAAGAGCAGGAGGCTGA and REV CCAGCATGCAGGTACTCAGA; *αCgrp* FOR CAGGCCTGAACAGATAACAGC and REV TGTGTCTTTCATCAGCCTTTCTT (Supplementary Fig. 2a). To monitor *CALCA* expression in HEK cells, the following primers were used: *CALCA* FOR TCAGCATCTTGGTCCTGTTG and REV CTGCACATAGTCCTGCACCA; *GAPDH* FOR CTGCACCACCAACTGCTTAG and REV ACAGTCTTCTGGGTGGCAGT. Gene expression was quantified as virtual copy number per housekeeping gene copy or as fold expression by the ΔΔCT method as indicated. *Gapdh* expression was used as the internal control.

Genome-wide expression analysis was performed using a Clariom D array kit (Thermo Fisher Scientific, Inc.) according to the manufacturer’s instructions. In brief, RNA quality was assessed by spectrophotometry (Nanodrop Technology, Inc.) and measurement of integrity (TapeStation 2200, Agilent Technologies, Inc.). The RIN range of the samples used for further assessment was 7.8–8.2. One hundred nanograms of total RNA from macrophage cultures was used as input for cRNA synthesis, and 15 µg of cRNA was subsequently used for cDNA synthesis. The cDNA was fragmented and labeled prior to microarray hybridization at 45 °C for 16 h. After washing and staining in an Affymetrix Fluidics Station 450 (Thermo Fisher Scientific, Inc.), the microarrays were scanned with a GeneChip Scanner 3000 7 G (Thermo Fisher Scientific, Inc.). Data analysis was performed in a Transcriptome Analysis Console v. 4.0.1.36 (Thermo Fisher Scientific, Inc.) employing default settings. Average fold change values were calculated using Tukey’s biweight mean algorithm. For all displayed genes, FDR-adjusted *P* > 0.1.

### Biochemical assays

Bone turnover markers were measured in serum samples by ELISA (RatLaps, BioVendor; RANKL, R&D; OPG, R&D). Cell culture supernatants and serum samples were analyzed for the concentrations of ProCT, CT, and CGRP by ELISA (Cusabio CSB-E10371m; LSBio LS-F23047; LSBio LS-F37469). Ionized calcium, inorganic phosphate, 1,25-dihydroxyvitamin D and FGF23 serum levels were determined by colorimetric assays and ELISA, as indicated (QuantiChrom Calcium Assay Kit, Fa. Bioassay Systems, #DICA-500; Stanbio Phosphorus Liqui-UV, Fa. Stanbio Laboratory, #0830; 1,25-Dihydroxyvitamin D EIA, Fa. Ids Immunodiagnosticsystems, #AC-62F1; FGF-23 ELISA Kit, Fa. KAINOS Laboratories Inc., TCY4000E).

### Western blot analysis

For western blot analysis, whole cells and thyroid samples were lysed in PBS with 0.1% Triton X-100 containing a protease and phosphatase inhibitor cocktail (Roche). Equal amounts of protein were separated on 12.5% SDS-polyacrylamide gels or on NuPAGE 4%–12% Bis-Tris protein gels and transferred to nitrocellulose or PVDF membranes (Hybond; GE Healthcare). After blocking with Tris-buffered saline containing 0.1% Tween 20 and 5% nonfat dry milk, membranes were incubated overnight at 4 °C with primary antibodies at a dilution of 1:250. The antibodies were directed against ProCT (LSBio, LS-C296040 or Cloud Clone Corp. #PAA689Mu01, as indicated in the figure legends), β-actin (Cell Signaling, #4967 S) and Gapdh (Cell Signaling, #2118). HRP-conjugated secondary antibodies (Dako Cytomation) or IRDye 800CW-conjugated antibodies (LI-COR) were used at a dilution of 1:2 000. Signals were quantified using Image Studio V5.2 software (LI-COR).

### Immunohistochemistry

Immunohistochemistry was performed on paraffin sections from the tibia. For immunohistochemical detection, sections were deparaffinized, rehydrated, and pretreated with Target Retrieval Solution (pH 9) overnight at 60 °C. The sections were incubated with a rabbit polyclonal anti-CD68 primary antibody from Boster (PA1518, 0.2 mg·mL^−1^, 1:200) overnight at 4 °C and were then incubated with a biotinylated secondary goat anti-rabbit IgG (1:200, Dako Cytomation). The avidin/biotin alkaline phosphate enzyme complex (ABC Kit, Vectastain, Vector) was applied for 30 min. Alkaline phosphatase activity was visualized using Permanent Red (Dako Cytomation) as the chromogenic substrate. Sections were counterstained with toluidine blue and mounted. Cells were manually counted using ImageJ in 4 representative images of the mid-diaphyseal area of tibia sections. To account for differences in bone marrow area between the different groups, the mineralized bone area was subtracted from the total measured bone marrow area. The results are expressed as Cd68-positive cells per mm^2^ bone marrow.

### Immunofluorescence

For localization of the CLR protein in cryo-embedded spine samples, nonconsecutive serial sections were used. For staining of CLR sections, sections were permeabilized with PBS/0.5% Triton X-100 for 10 min and washed with PBS/0.25% Triton prior to blocking in 3% BSA/5% donkey serum with 0.1% Triton. After blocking, the sections were incubated overnight with an anti-CLR primary antibody (rabbit) (1:200, bs-1860R-TR, Bioss Antibodies). Next, the sections were washed in PBS, incubated with the secondary antibody (1:400, anti-rabbit-Cy3, 711-165-152, Dianova) and mounted in Fluoromount-G with DAPI (Thermo Fisher). Images were acquired using a Keyence BZ-X810 microscope (Keyence) and Keyence BZ-X Analysis software (Keyence).

### Flow cytometric analysis

Bone marrow of iPTH-treated mice was extracted and filtered. The resulting single-cell suspensions were incubated with antibody cocktails, and analysis was performed with an LSR Fortessa flow cytometer and Diva software (BD Pharmingen). Antibodies are listed in the antibody reference list (Supplementary Table 1). Gating was performed as described previously^66^ (Supplementary Fig. 8).

### Biomechanical testing

Mechanical testing was performed on explanted femora. A BZ2.5 material testing machine and testXpert software (both from Zwick Roell) were utilized for destructive three-point bending tests as described previously.^67^ In brief, support bars with a 7 mm spacing were used to hold the femora while a tip was lowered medially at a constant rate of 2 mm·min^−1^. The distance and force were recorded at 100 Hz until failure. All parameters were calculated with testXpert software based on these data.

### Statistical analyses

For two-group comparisons, data were analyzed by two-tailed Student’s *t* test using GraphPad Prism software. For multiple-group comparisons, one-way or two-way ANOVA followed by Tukey’s post hoc test was applied as indicated. Unless stated otherwise, all data are shown as the means ± SDs. *P* < 0.05 was considered statistically significant. Due to loss or destruction of samples, the number of available samples for analyses varied and is indicated with individual data points for each experiment.

## Supplementary information

Baranowsky Supplementary information
