# Supplementary material for: Procalcitonin is expressed in osteoblasts and limits bone resorption through inhibition of macrophage migration during intermittent PTH treatment
Source: Bone Res. 2022 Jan 27;10:9. doi: 10.1038/s41413-021-00172-y (PMC8795393; doi:10.1038/s41413-021-00172-y)
Supplement: Supplementary file 1 — Baranowsky Supplementary information [file 41413_2021_172_MOESM1_ESM.docx]

**Supplementary information**

Procalcitonin is expressed in osteoblasts and limits bone resorption through inhibition of macrophage migration during intermittent PTH treatment

Anke Baranowsky^1,2,#^, Denise Jahn^3,4,#^, Shan Jiang^1^, Timur Yorgan^2^, Peter Ludewig^5^, Jessika Appelt^3,4^, Kai K. Albrecht^4^, Ellen Otto^3,4^, Paul Knapstein^1^, Antonia Donat^1^, Jack Winneberger^5^, Lana Rosenthal^2^, Paul Köhli^3,4^, Cordula Erdmann^1^, Melanie Fuchs^3,4^, Karl-Heinz Frosch^1^, Serafeim Tsitsilonis^3,4^, Michael Amling^2^, Thorsten Schinke^2^ and Johannes Keller^1,6,*^

^1)^ Department of Trauma and Orthopedic Surgery, University Medical Center Hamburg-Eppendorf, Hamburg 20246, Germany

^2)^ Department of Osteology and Biomechanics, University Medical Center Hamburg-Eppendorf, Hamburg 20246, Germany

^3)^ Center for Muskuloskeletal Surgery, Charité-Universitätsmedizin Berlin, Berlin 13353, Germany

^4)^ Julius Wolff Institute for Biomechanics and Musculoskeletal Regeneration, Charité-Universitätsmedizin Berlin, Berlin 13353, Germany

^5)^ Department of Neurology, University Medical Center Hamburg-Eppendorf, Hamburg 20251, Germany

^6)^ Berlin Institute of Health, Berlin 10178, Germany

^#^equal contribution

^*^corresponding author: j.keller@uke.de


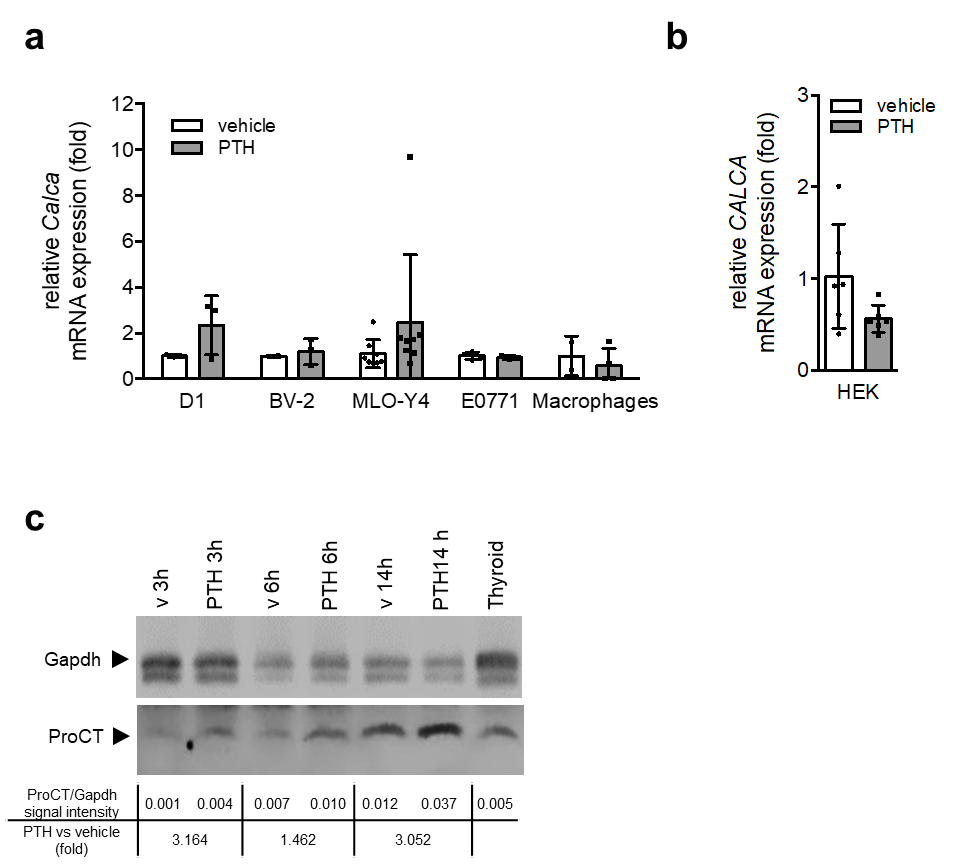


**Supplementary Figure 1.** (**a**) Relative gene expression of *Calca* and (**b**) human *CALCA* in the indicated cell lines and primary cells following stimulation with 10^-7^ M PTH for 6 hours. n = 3-6 independent cultures per group with the exception of MLO-Y4 cells, where n = 8 cultures were used (one-way Anova followed by Tukey’s post-hoc test). (**c**) Western blot monitoring ProCT expression in calvaria-derived osteoblasts treated with PTH (10^-7^M) for the indicated durations. The expected band sizes of ProCT (17 kDa; LSBio antibody) and Gapdh (37 kDa) are indicated. The ratios of ProCT/Gapdh signal intensity and the increased ProCT production induced by PTH at each time point (fold) are displayed below.

**Supplementary Figure 2.** (**a**) Schematic overview of tissue-specific alternative splicing of the *Calca* gene and the resulting signaling peptides. Whereas CT exclusively signals through the CTR, ProCT and αCGRP primarily bind to CLR/RAMP1 receptor complex. (**b**) Tabular overview of the applied mouse models with focus on the inactivated signaling transduction pathways.

**
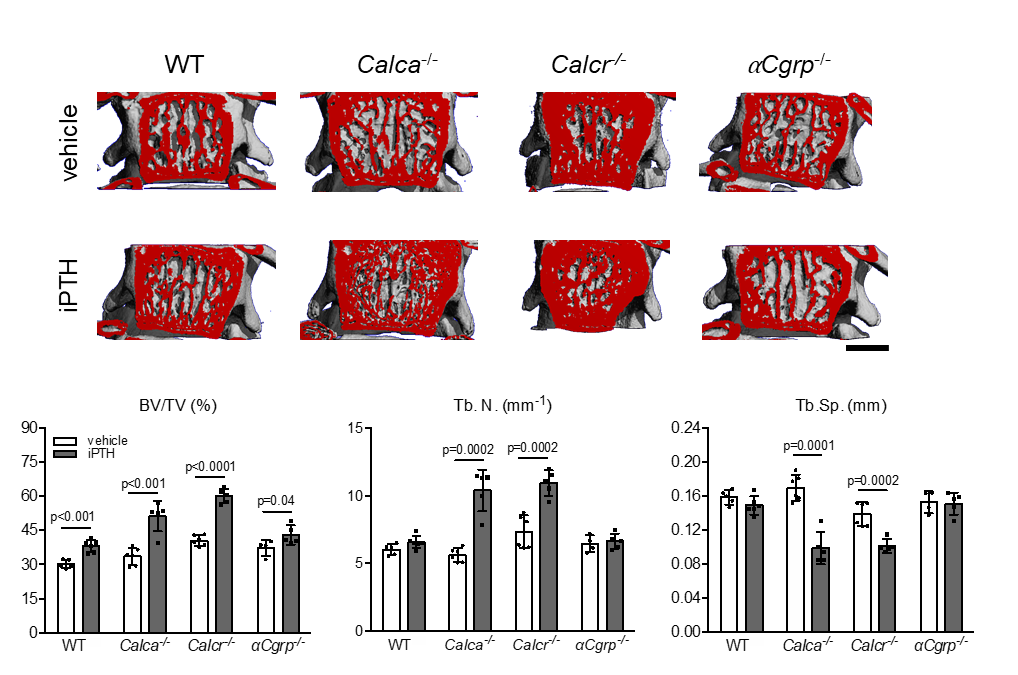
Supplementary Figure 3.** Representative μCT images of the 12^th^ thoracic vertebra of the indicated groups following 4 weeks of treatment with iPTH (100 μg/kg) or vehicle. Scale bar = 1 mm. The quantification of the trabecular bone volume per tissue volume (BV/TV), trabecular numbers (Tb.N.) and trabecular separation (Tb.Sp.) is depicted below. n = 4-6 mice per group (one-way Anova followed by Tukey’s post-hoc test).

**
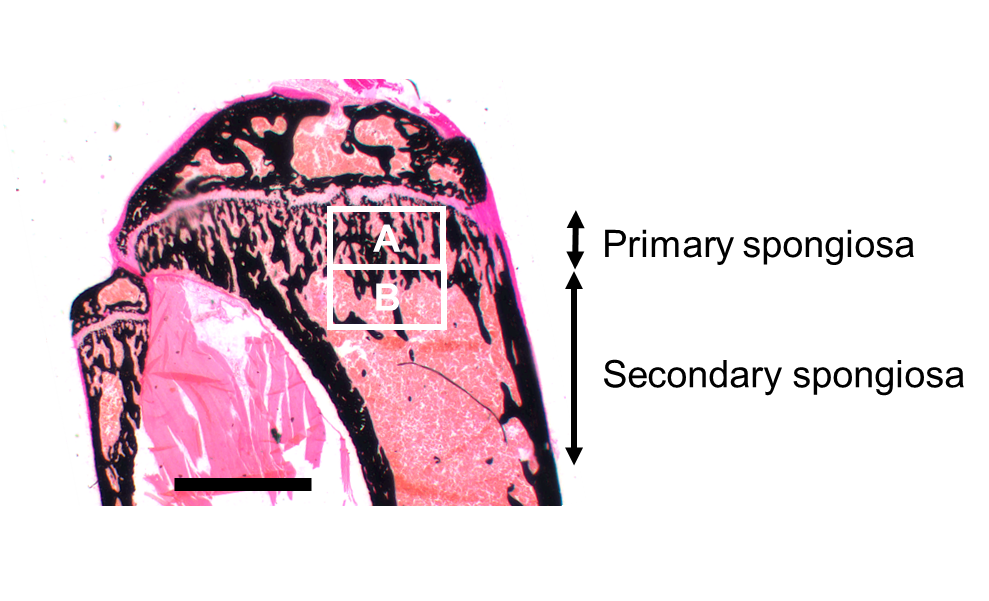
**

**Supplementary Figure 4.** Histomorphometric approach of the proximal tibia. Histomorphometry of primary spongiosa was performed by manually marking ROI A (400 μm x 200 μm), delimited by the lower border of the mineralized growth plate and the distal end of primary spongiosa. For quantification of the secondary spongiosa, a rectangular ROI B with the same dimensions as ROI A and not overlapping with the two cortices was used. Structural trabecular parameters for each sample were calculated as the mean of ROI A and ROI B. Scale bar = 500 μm.


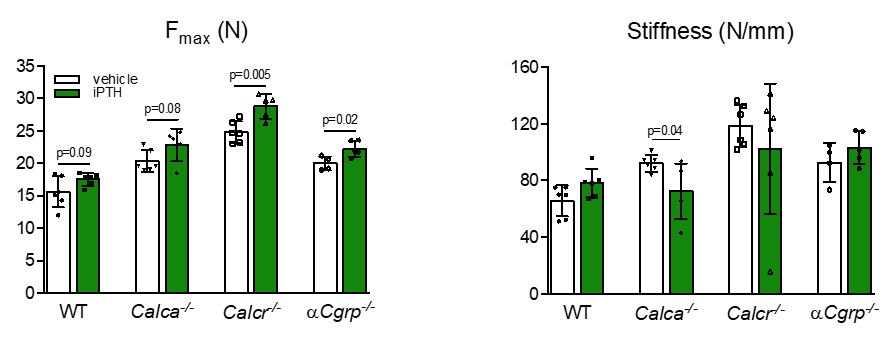


**Supplementary Figure 5.** Biomechanical testing of the femora from the indicated groups following 4 weeks of treatment with iPTH (100 μg/kg) or vehicle. n = 5-6 mice per group (one-way Anova followed by Tukey’s post-hoc test).

**
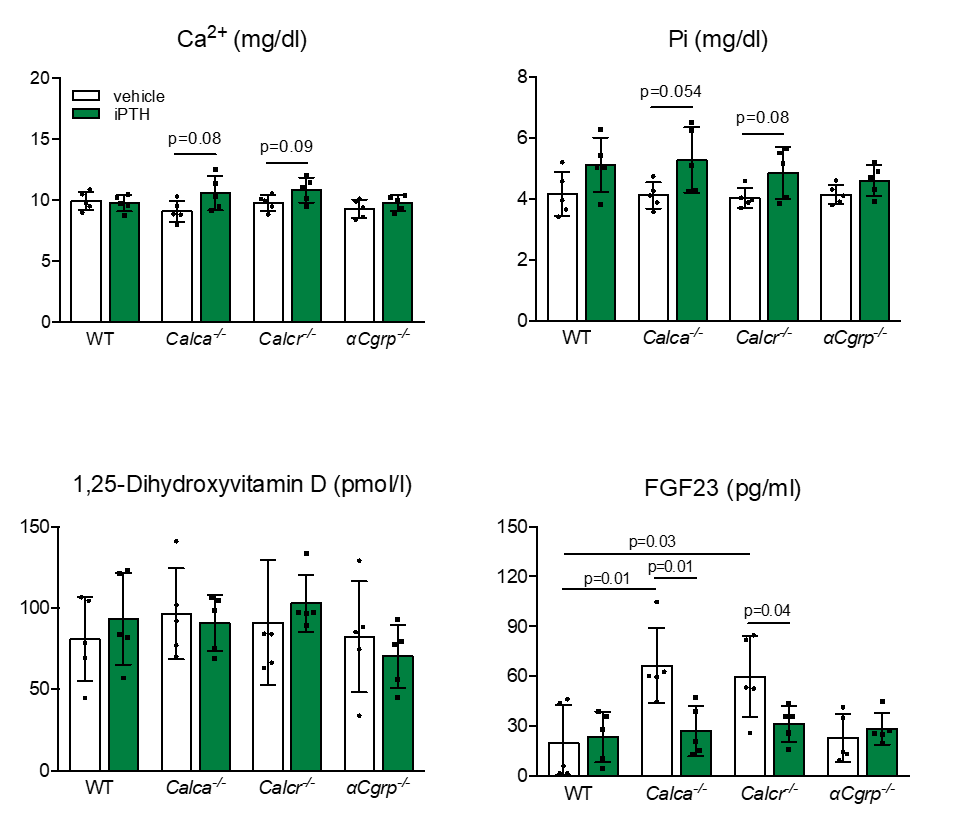
Supplementary Figure 6.** Serum concentrations of the indicated minerals and calciotropic hormones in WT and mutant mice 2h after a single iPTH or vehicle injection, measured by colorimetric assays or ELISA, respectively. n = 5 mice per group (two-way Anova followed by Tukey’s post-hoc test).

**
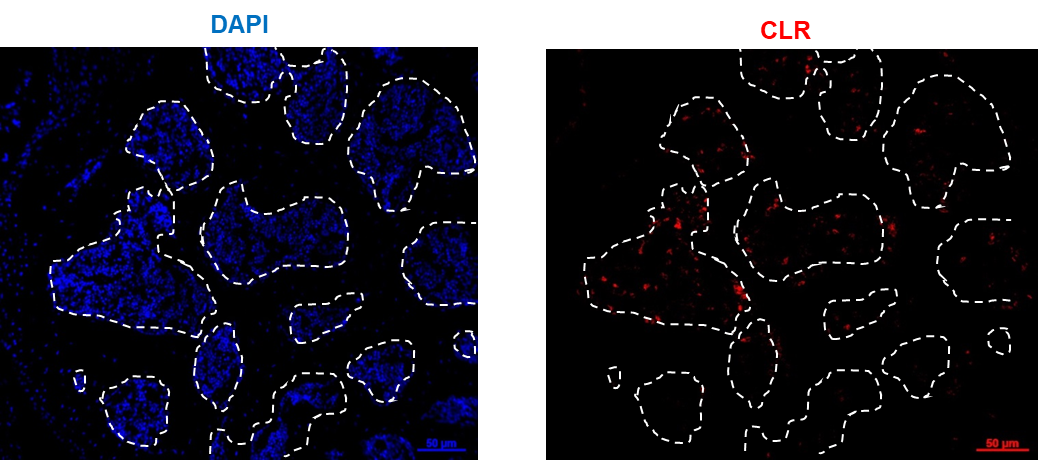
**

**Supplementary Figure 7.** Representative immunofluorescent stainings (individual channels) of intact WT spine sections using a CLR-specific antibody.


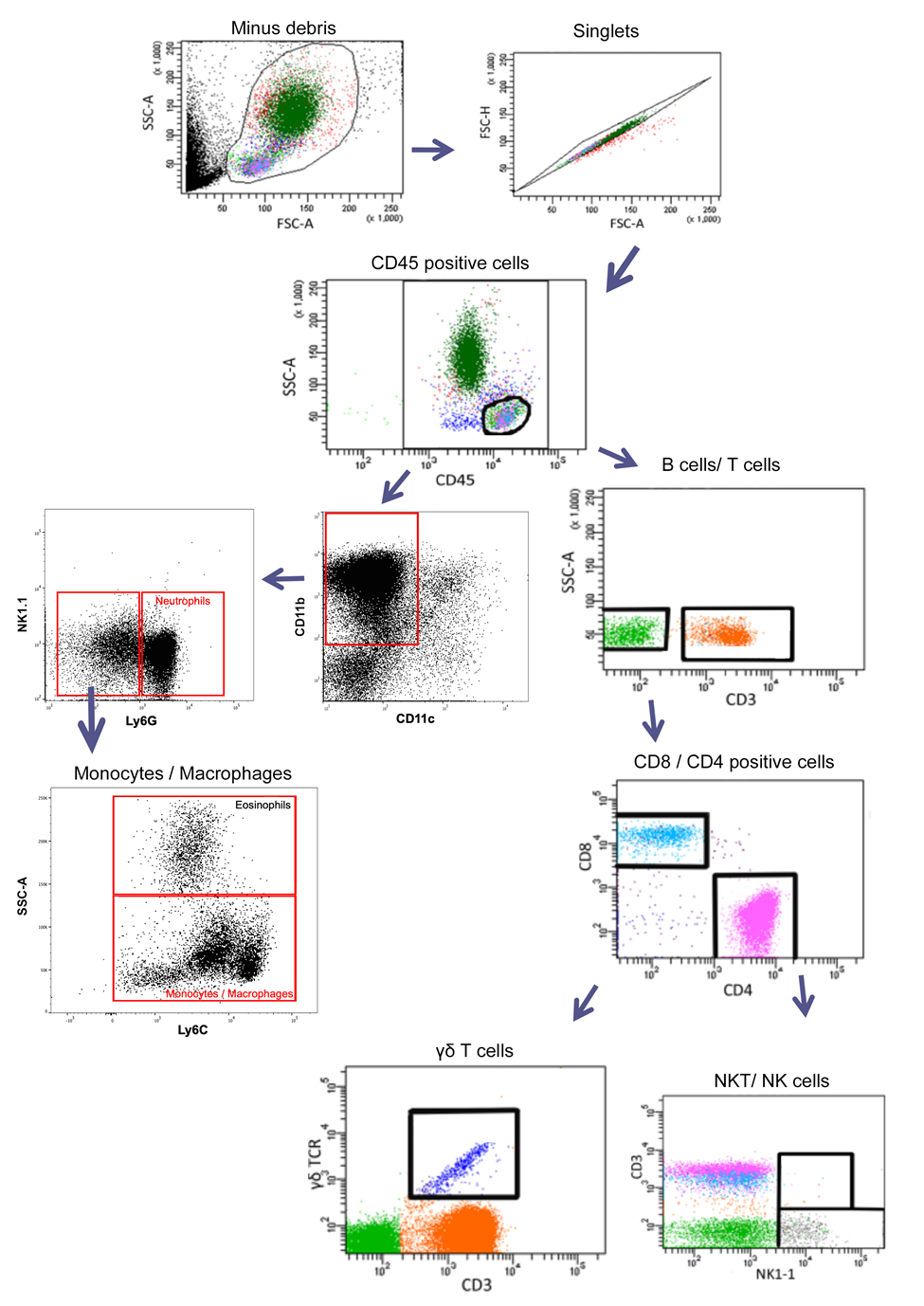


**Supplementary Figure 8.** Applied gating strategy. Myeloid cell populations were identified with a multicolor flow cytometry panel including the markers CD11b, CD11c, Ly6C, Ly6G, NK1.1, CD3, CD4, CD8, B220. Macrophages/monocytes were detected as Ly6C^Lo–neg^Ly6G^-^SSC^lo^ and Ly6C^+^Ly6G^–^SSC^lo^ cells, neutrophils as Ly6G^Hi^CD11b^+^cells, eosinophils as Ly6C^Lo–neg^ Ly6G^–^SSC^Hi^ cells. Lymphocytes were gated out using CD3, CD4, CD8, and B220.


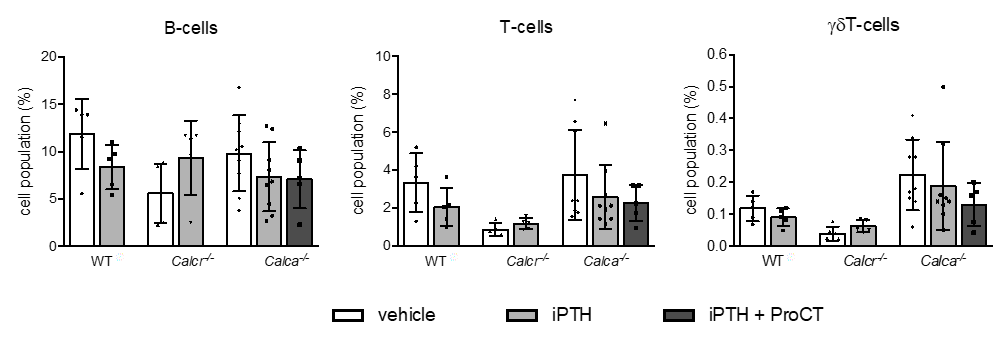


**Supplementary Figure 9.** Flow cytometry analyses of indicated cell populations in flushed bone marrow from mice of the indicated genotypes following 4 weeks of iPTH (100 μg/kg) and/or ProCT (10 μg/kg). B-cells were identified with the marker B220, T-cells with the marker CD3, CD4, CD8, the γδT-cells with the TCR γδ. n = 4-9 mice per group (one-way Anova followed by Tukey’s post-hoc test).

**Supplementary Table 1. Antibody reference list**

| **Primary antibodies for FACS** | **Origin** | **Dilution** | **Company** |
| --- | --- | --- | --- |
| Alexa Fluor® 488 anti-mouse CD11c Antibody | Armenian Hamster | 1:100 | Biolegend |
| Alexa Fluor® 700 anti-mouse Ly-6G Antibody | Rat | 1:100 | Biolegend |
| APC/Cyanine7 anti-mouse CD45 Antibody | Rat | 1:100 | Biolegend |
| Brilliant Violet 421™ anti-mouse TCR γδ Antibody | Armenian Hamster | 1:100 | Biolegend |
| Brilliant Violet 570™ anti-mouse CD4 Antibody | Rat | 1:100 | Biolegend |
| Brilliant Violet 650™ anti-mouse CD8a Antibody | Rat | 1:100 | Biolegend |
| Brilliant Violet 785™ anti-mouse/human CD45R/B220 Antibody | Rat | 1:100 | Biolegend |
| PE-CF594 Rat Anti-CD11b | Rat | 1:300 | BD Biosciences |
| PE-Cy™7 Mouse Anti-Mouse NK-1.1 | Mouse | 1:50 | BD Biosciences |
| PerCP/Cyanine5.5 anti-mouse Ly-6C Antibody | Rat | 1:100 | Biolegend |
| Purified Rat Anti-Mouse CD16/CD32 (Mouse BD Fc Block™) | Rat | 1:100 | BD Biosciences |
| V500 Syrian Hamster anti-Mouse CD3e | Syrian Hamster | 1:100 | BD Biosciences |
